# Language assessment and treatment in the last decade

**DOI:** 10.1590/S1980-57642014DN83000001

**Published:** 2014

**Authors:** Leticia Mansur, Karin Zazo Ortiz, Jed A. Meltzer

**Affiliations:** Guest Editors

As guest editors, we are happy to release this volume of **Dementia *&* Neuropsychologia**, focused on Language assessment and treatment.

The **Views *&* Reviews** section brings two texts. *Evolution of Language Assessment in Patients with Neurological Acquired Disorders in Brazil* presents a historical perspective and an overview of the transformations undergone by language evaluation of patients with acquired neurological damage in Brazil. *Transcranial brain stimulation for post-stroke aphasia rehabilitation* discusses consensus and controversies about modern techniques for rehabilitation of language disorders arising from focal neurological lesions.

In the **Original Articles** section, we have 11 articles, seven focused on evaluation and four on rehabilitation. Adaptation and cross-cultural validation to the Portuguese language were the aims in *Semantic memory for actions and Adaptation of the CAPPA to the Portuguese*. Methods of language analysis for healthy individuals are presented in *Automatic Classification of Written Descriptions*. Oral and written language evaluations of individuals with focal lesions were also examined in *Acquired dysgraphia in adults and Inferential abilities in patients with right hemisphere damage*. Diffuse lesions were the topic of *Naming performance in bilingual speakers with dementia and Discourse of Patients with mild to moderate Alzheimer's disease*. The issue of rehabilitation was addressed in a systematic review - *Rehabilitation of lexical and semantic communicative impairments* and in two interventional studies in dementia: *Discourse intervention in Alzheimers disease and Interdisciplinary therapy in dementia*.

Finally, in the session of **Case Reports** different methods in the rehabilitation of agrammatism were presented: in degenerative disease, *Brief intervention for agrammatism in Primary Progressive Nonfluent Aphasia*, and in aphasia due to vascular lesion, *Sentence production and rehabilitation of agrammatism*.

We highlight the publication of a case study awarded by ACHÉ Laboratory in an initiative of Prof. Dr. Ricardo Nitrini and Dr. Sonia Brucki. This case study was aimed at differential diagnosis between *Logopenic aphasia and Alzheimer's*.

We hope this issue will contribute to knowledge on Recent Changes in Assessment and Intervention in Neurological Disorders of Language.

**Leticia Mansur** **Karin Zazo Ortiz** **Jed A. Meltzer** Guest Editor

